# Inhibitory Effects of a Variety of Aldehydes on *Amaranthus tricolor* L. and *Echinochloa crus-galli* (L.) Beauv.

**DOI:** 10.3390/molecules23020471

**Published:** 2018-02-21

**Authors:** Nawasit Chotsaeng, Chamroon Laosinwattana, Patchanee Charoenying

**Affiliations:** 1 Department of Chemistry, Faculty of Science, King Mongkut’s Institute of Technology Ladkrabang, Bangkok 10520, Thailand; patchanee.ch@kmitl.ac.th; 2 Department of Plant Production Technology, Faculty of Agricultural Technology, King Mongkut’s Institute of Technology Ladkrabang, Bangkok 10520, Thailand; klchamro@kmitl.ac.th

**Keywords:** inhibitory, herbicidal, allelopathy, aldehydes, Chinese amaranth, barnyardgrass

## Abstract

Thirty-seven commercial aldehydes containing aliphatic chains and aromatic rings as well as heteroaromatic rings were evaluated for their inhibitory activities against Chinese amaranth (*Amaranthus tricolor* L.) and barnyardgrass (*Echinochloa crus-galli* (L.) Beauv). Polysorbate 80 (Tween^®^ 80) was used as a surfactant and the research was preliminarily conducted at 400 μM of all aldehydes. Among these aldehydes, (*E*)-cinnamaldehyde (**7**) showed the greatest inhibitory effect on seed germination, shoot and root growth of Chinese amaranth by 54.55%, 75.53%, and 85.13% respectively. Similarly, (*E*)-crotonaldehyde (**5**), a related α,β-unsaturated aldehyde, inhibited the germination and seedling growth of the tested species at a high percentage. Apart from these two unsaturated aldehydes, no other aliphatic aldehydes had a harmful effect on Chinese amaranth. In terms of benzaldehyde (**6**), it had no effect on the tested plant; however, many of its derivatives displayed some inhibitory activity. Furthermore, for the ten common heteroaromatic aldehydes, picolinaldehyde (**32**) had a high inhibitory effect on Chinese amaranth which closely related to the effect of (*E*)-crotonaldehyde (**5**) and (*E*)-cinnamaldehyde (**7**), whereas, other heteroaromatic aldehydes showed lower effects. In the case of a monocot plant, barnyardgrass, no tested aldehydes reduced seed germination, however, (*E*)-cinnamaldehyde (**7**), 2,4,6-trimethoxybenzaldehyde (**16**) and 4-(dimethylamino)benzaldehyde (**24**) could inhibit the seedling growth of the plant with low to moderate levels. The herbicidal effects of the most active aldehydes were then further investigated in order to find the minimum concentration of these aldehydes suppressing the germination and growth of the tested plants. At concentrations as low as 50–100 μM some aldehydes could inhibit the seedling growth of the tested species. The structure-activity relationship (SAR) study reported here demonstrates the chemical clues governing the inhibitory activity of aldehydes which could be utilized in the development of highly effective herbicides in the near future.

## 1. Introduction

Weeds are a major problem on the yield of agricultural crops. They compete with crop plants for water, nutrients, space or even sunlight, causing the crop plants to grow slowly or even die [1,2]. Therefore, in order to achieve high crop production and yields, it is crucially important to control these unwanted species. In general, weed management practices vary widely depending upon climatic and environmental conditions as well as weed species. Overall, the main methods of weeding are manual, mechanical, chemical and biological controls respectively [3,4,5]. Among those methods, chemical control is one of the most popular procedures. Moreover, the use of natural compounds or allelochemicals for weeding has been studied extensively [3,6,7,8,9,10,11,12,13,14,15]. Allelochemicals are substances released from plants or microorganisms to control the growth of other plants or other small organisms [14,16]. Usually, it is believed that natural products or naturally-occurring compounds are safer than synthetic chemicals, comparably easy to decompose and environmentally friendly [12,13,17]. Also, they could be conveniently used in a form of both pure chemical and crude extract. Xanthoxyline and (±)-odorine are two examples of such allelochemicals which were successfully isolated in our research group from Makhwaen (*Zanthoxylum limonella* Alston) fruits and Prayong (*Aglaia odorata* Lour.) leaves [18,19]. Both natural compounds inhibited well the seed germination and seedling growth of tested plants, Chinese amaranth, and barnyardgrass. In addition to those examples mentioned above, there are numerous reports showing the diverse groups of allelochemicals or natural products which are used as herbicides [6,13,14,20,21,22,23,24]. For examples, fatty acids, essential oil, amino acids, peptides, alkaloids, flavonoids and phenolics etc. These compounds could inhibit seed germination and seedling growth of tested weeds, algae and microorganisms. Although, the herbicidal properties of these chemicals have been studied for many years, a thorough investigation of allelopathic potentials of each specific chemical class is still required. Commonly, purification and identification of bioactive natural products are time-consuming and quite expensive, especially, with the limited natural resources [9,14]. Frequently, only minute quantities of allelochemicals are obtained. Moreover, several of them have complex chemical structures which lead to expensive, long and difficult synthetic procedures to access. Their structure-activity relationship (SAR) studies, therefore, are difficult to accomplish.

**Figure 1:**
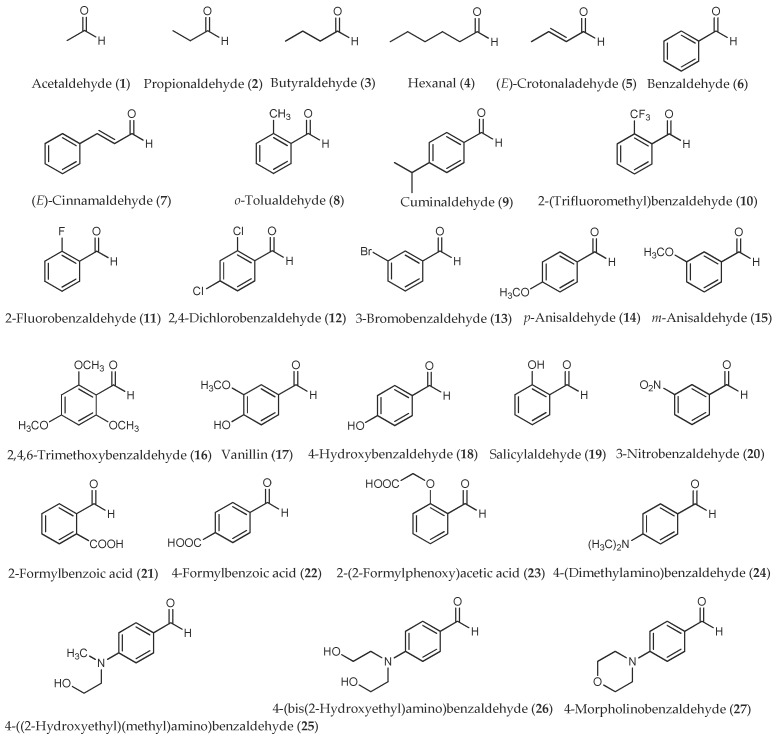
Aliphatic and aromatic aldehydes used in this study.

**Figure 2:**
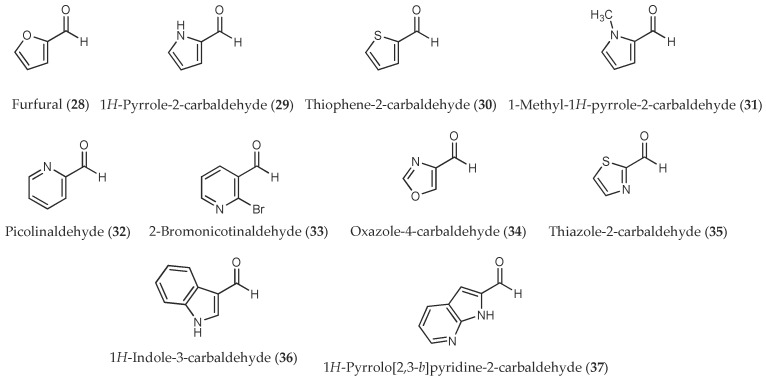
Heteroaromatic aldehydes used in this study.

In this study we are interested in investigating the herbicidal activities of a variety of aldehydes (both aliphatic and aromatic aldehydes (Figure 1) as well as heteroaromatic aldehydes (Figure 2)) on seed germination and seedling growth of two tested plants, Chinese amaranth (*Amaranthus tricolor* L.) and barnyardgrass (*Echinochloa crus-galli* (L.) Beauv). Both species were chosen as representatives of dicot and monocot plants, respectively. We selected an aldehyde chemical class since there are several reports indicating that natural and synthetic aldehydes [25,26,27,28,29,30,31,32,33,34,35,36] or crude extracts containing aldehydes [37,38,39,40,41,42,43,44] could interrupt the germination, growth, and development of plants, algae, and microorganisms. Furthermore, numerous aldehydes are commercially available in pure form which can be directly purchased. Positive results of the current research could be applied in the development of new and highly reactive herbicides.

## 2. Results and Discussion

### 2.1. Inhibitory Effects of Thirty-Seven Aldehydes on Germination and Seedling Growth of Chinese Amaranth

**Figure 3:**
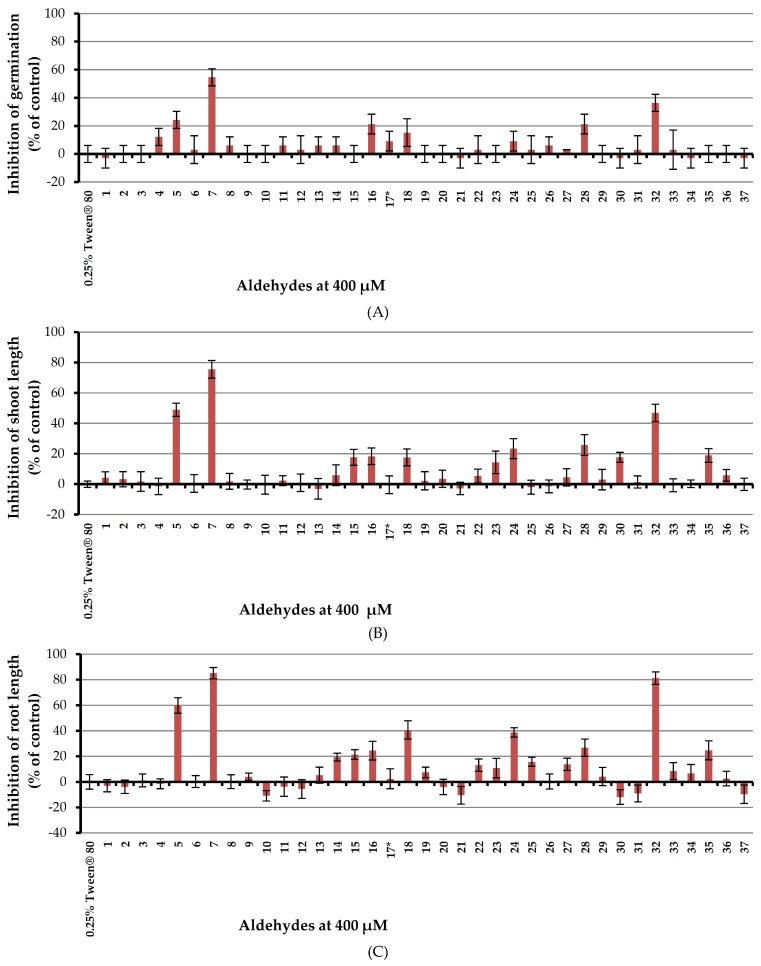
Inhibitory effects of aqueous solutions of thirty-seven aldehydes (at 400 µM) on seed germination (**A**), shoot length (**B**), and root length (**C**) of Chinese amaranth. A 0.25% (*v*/*v*) aqueous solution of Tween^®^ 80 was used as a control. * Result from a previous study [45], the effect of vanillin (**17**) on Chinese amaranth.

In the preliminary investigation, Chinese amaranth was selected as a representative dicotyledon. According to our previous study [45] 0.25% (*v*/*v*) aqueous solution of Tween^®^ 80 (Sigma-Aldrich, Singapore) was used as a surfactant. A basic structure-activity relationship (SAR) study was performed by using three groups of aldehydes namely; aliphatic, aromatic and heteroaromatic respectively. The results revealed that (Figure 3), four aliphatic aldehydes containing 2–6 carbon atoms (acetaldehyde (**1**), propionaldehyde (**2**), butyraldehyde (**3**) and hexanal (**4**)) had no effect on germination and seedling growth of Chinese amaranth. Fortunately, unsaturated aliphatic aldehyde such as (*E*)-crotonaldehyde (**5**) could inhibit seed germination, shoot, and root elongation by 24.24, 48.89, and 59.88% respectively. In comparison with butyraldehyde (**3**) which is similarly composed of four carbon atoms, (*E*)-crotonaldehyde (**5**) showed much higher inhibitory activity. Moreover, the size of a substituent attached to an α,β-unsaturated part could possibly determine the inhibitory effect of the compound. Accordingly, (*E*)-cinnamaldehyde (**7**) showed a greater herbicidal effect against Chinese amaranth than (*E*)-crotonaldehyde (**5**) did. At a concentration of 400 μM, (*E*)-cinnamaldehyde (**7**) inhibited seed germination, shoot and root length of the tested plant by 54.55, 75.53, and 85.13% respectively, which indicates the most reactive chemical among all aldehydes.

In terms of aromatic aldehydes, both benzaldehyde (**6**) and its derivatives bearing alkyl substituents (aldehydes **8**–**9**) and halogen substituents (aldehydes **10**–**13**) had no inhibitory effect on Chinese amaranth. However, for the derivatives containing methoxy-substituent (compounds **14**–**16**) it turned out that position and number of the substituents affected the activity which meta-position (*m*-anisaldehyde **15**) expressed higher effect than para-position (*p*-anisaldehyde **14**) and tri-substituted (2,4,6-trimethoxybenzaldehyde **16**) had a greater effect than monosubstituted (aldehydes **14**–**15**).

For derivatives having hydroxyl-substituents (compounds **17**–**19**), the *para*-position (4-hydroxybenzaldehyde **18**) seemed to have greater effect of herbicidal potential than the *ortho*-position (salicylaldehyde **19**). Nevertheless, vanillin (**17**) containing both a *para*-hydroxyl group and a *meta*-methoxy group had very low phytotoxicity against the tested plant. 3-Nitrobenzaldehyde (**20**), likewise, had no effect on the tested plant. Next, the effect of a carboxyl substituent (compound **21**–**23**) was investigated and it appeared that the substitution on the chain outside the benzene ring (aldehyde **23**) had more effect than the substitution on the ring (aldehydes **21**–**22**). Among aldehydes with amino-substituent (compounds **24**–**27**), 4-(dimethylamino)benzaldehyde (**24**) exhibited a moderate inhibitory effect on the tested plant. In the case of heteroaromatic aldehydes **28**–**37**, picolinaldehyde (**32**) inhibited seed germination, shoot and root growths of Chinese amaranth by 36.36, 46.87, and 81.28% respectively. The derivatives **28**, **30** and **35** showed some activities, but other heteroaromatics had no effect.

**Figure 4:**
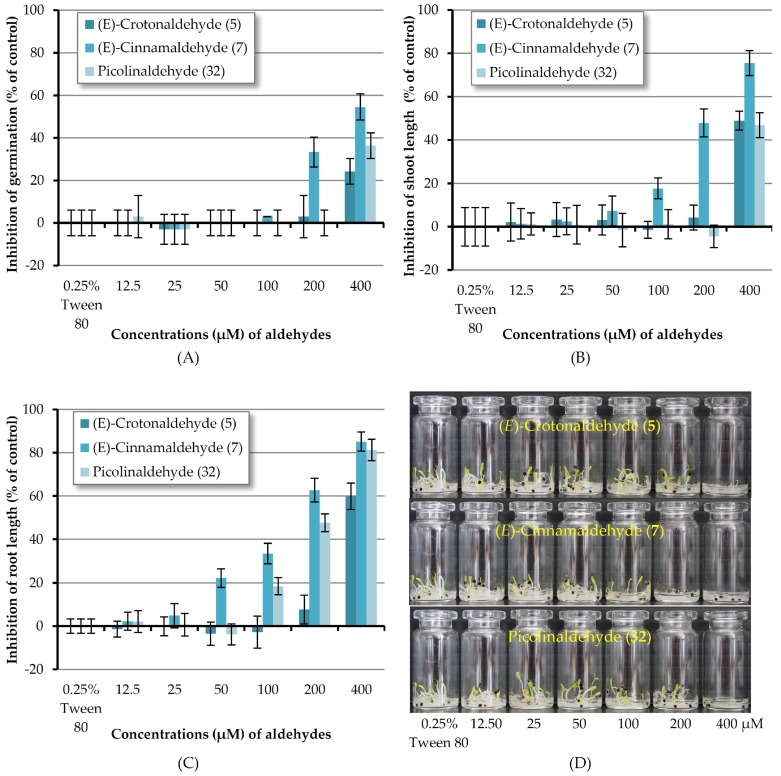
Inhibitory effects of aqueous solutions of three aldehydes (at 12.5–400 µM) on seed germination (**A**), shoot length (**B**), and root length (**C**) of Chinese amaranth. A 0.25% (*v*/*v*) aqueous solution of Tween® 80 was used as a control. (**D**) Chinese amaranth in small vials.

According to the results mentioned above, (*E*)-crotonaldehyde (**5**), (*E*)-cinnamaldehyde (**7**) and picolinaldehyde (**32**) are the three most reactive chemicals towards Chinese amaranth. In order to know the minimum molar concentration at which all three compounds could suppress the germination and seedling growth of the tested dicot, these substances were then investigated at concentrations of 12.5–400 μM (Figure 4). It revealed that at 200 μM only (*E*)-cinnamaldehyde (**7**) could inhibit seed germination of the tested plant. Aldehyde **7** at the other concentrations and aldehydes **5** and **12** at 12.5–200 μM showed no effect. In terms of shoot length, although at 400 μM, aldehyde **5** and **32** could inhibit shoot growth, at 12.5–200 μM these two substances had no adverse effect on shoot length. (*E*)-Cinnamaldehyde (**7**), however, could inhibit shoot length at the concentrations as low as 50 μM. In the case of root development, at concentrations lower than 400 μM, (*E*)-crotonaldehyde (**5**) had no effect on root growth. Picolinaldehyde (**32**) could inhibit root length at the concentrations down to 100 μM. At concentrations of 50, 100 and 200 μM, the most reactive substance, (*E*)-cinnamaldehyde (**7**), inhibited root length by 22.18, 33.52, and 62.69% respectively.

The results stated above reveal the importance of an unsaturated structure on the herbicidal activity of aldehydes. This is consistent with numerous reports on the effects of some polyunsaturated aldehydes (PUAs) on diatoms, planktons, and algae [31,46,47,48,49,50,51,52]. For example, Casotti and coworkers [52] investigated the effects of three diatom-produced PUAs, 2*E*,4*E*-decadienal, 2*E*,4*E*-octadienal and 2*E*,4*E*-heptadienal, on six phytoplankton. The result showed that the reduction of growth rate of tested plankton was concentration-dependent and species-specific. Also, the longer-chained aldehydes had stronger effects on the plankton growth than the shorter-chained aldehydes which is in agreement with our result that (*E*)-cinnamaldehyde (**7**) showed greater adverse effect than (*E*)-crotonaldehyde (**5**). Vaughn and Spencer [53] examined the inhibitory effect of some naturally-occurring aromatic aldehydes and thymol on potato tuber sprouting and found that most tested compounds inhibited sprouting of tubers exposed up to 10 days. Moreover, direct application of 1% cinnamaldehyde (**7**) and 10% benzaldehyde (**6**) completely suppressed sprouting for 14 days after treatment without apparent tuber damage. Apart from PUAs, some unsaturated aldehydes are also found in reactive fractions of plant crude extracts. For instance, Song and coworkers [42] reported the allelopathic effects of crude extracts from the green peel of *Juglans mandshurica* Maxim. on three plants; *Brassica chinesis*, *Raphanus sativus*, and *Medicago sativa*. It was revealed that the alcohol extract and its ethyl acetate soluble fraction showed good inhibitory activity. After GC-MS analysis of the extracts it uncovered that aside from a major allelochemical component, juglone, some aldehydes such as 4 butoxybenzaldehyde, 5-(hydroxymethyl)-2-furancarboxaldehyde and 4-hydroxy-2-methoxycinnamaldehyde were also found in the active fractions.

Others have shown that (*E*)-cinnamaldehyde (**7**) also has antimicrobial activity [26,35,54,55]. For example, Zhang and coworkers [35] studied the structure-activity relationships (SAR) of cinnamaldehyde (**7**) and eugenol derivatives against two plant pathogenic fungi, *Rhizoctonia solani* and *Fusarium oxysporum*. It displayed that many derivatives showed good activities against both fungi. Interestingly, the fungicidal potential of cinnamaldehyde derivatives could be related to conjugated double bond and the length of CH chain outside the ring. Moreover, the authors suggested that the presence of the lipophilic part would be influent on the toxicity of phenylpropenes.

Regarding detrimental effects of heteroaromatic aldehydes, among those tested substances, furfural (**28**) and picolinaldehyde (**32**) seemed to have the greatest inhibition on Chinese amaranth. The reason behind this is still unclear; however, the allelopathic effects of natural products containing heteroaromatic parts have been extensively documented [56,57,58,59,60,61,62,63,64,65,66,67]. For examples of pyridyl and furanyl bearing compounds, Rizvi and coworkers [64] investigated the alellopathic activity of a pyridine containing alkaloid, nicotine, on maize (*Zea mays*) and rice (*Oryza sativa*) and found that nicotine adversely affected the germination, radicle and plumule length and seedling vigor of rice. On the other hand, it favorably affected the growth of maize by increasing the height, specific leaf weight, and chlorophyll content. Komai and coworkers [61] isolated a plant growth inhibitor, perilla ketone, from Egoma plant (*Perilla frutescens* var. *japonica*) and investigated its inhibitory effect on lettuce (*Lactuca sativa* L. c.v. new york.) and large crabgrass (*Digitaria adsendens* Henr.). Apparently, this ketone inhibited the radicle elongation of the tested plants at concentrations of 50–100 ppm. However, it did not inhibit seed germination of lettuce. In 2017, Chahal and coworkers [62] determined the chemical compositions of root and rhizosphere soil extracts of allelopathic plant, Marigold (*Tagetes patula* L.) using GC-MS analysis method. Twenty-five and twenty-seven compounds were identified in the two fractions. 5-Hydroxymethylfurfural was one of the major components in the methanol root extract which comprised of 21.81%. Eventually, the authors suggested that those leached compounds would be responsible for the allelopathic potential of this plant.

### 2.2. Effects of Tween^®^ 80 Surfactant on Germination and Seedling Growth of Barnyardgrass

**Figure 5:**
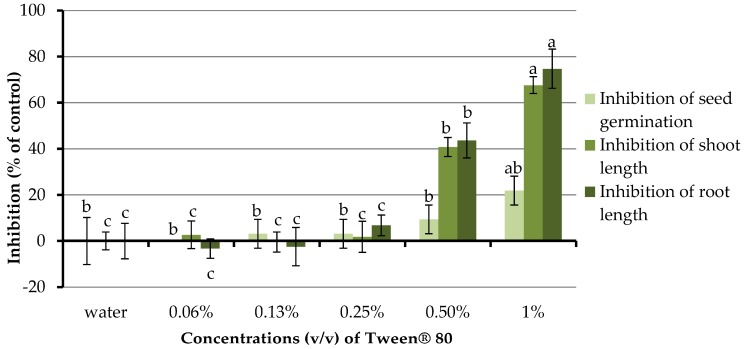
Effects of Tween^®^ 80 surfactant (at concentrations of 0.06–1.00% (*v*/*v*)) on the germination and growth of barnyardgrass. Distilled water was used as a control treatment. Means with the same letters in the graph are not significantly different at *p* ≤ 0.05 level.

Although Tween^®^ 80 helps emulsifying organic compound in an aqueous solution, it could also affect the germination and growth of plants depending upon the applied concentrations [45]. Therefore, the minimum quantity of this surfactant that influences the germination and growth of barnyardgrass have to be evaluated. The concentrations of the aqueous solution of Tween^®^ 80 being investigated were 0.06–1.0% and distilled water was used as a control treatment (Figure 5). Clearly, at concentrations of 0.06–0.25% Tween^®^ 80 had no significant effect on germination and seedling growth of barnyardgrass. However, at concentrations of 0.5 and 1.0%, this surfactant highly inhibited the germination and development of barnyardgrass. Therefore, Tween^®^ 80 at a concentration of 0.25% was chosen to use as a proper surfactant in the next section.

### 2.3. Inhibitory Effects of Thirty-Seven Aldehydes on Germination and Seedling Growth of Barnyardgrass

**Figure 6:**
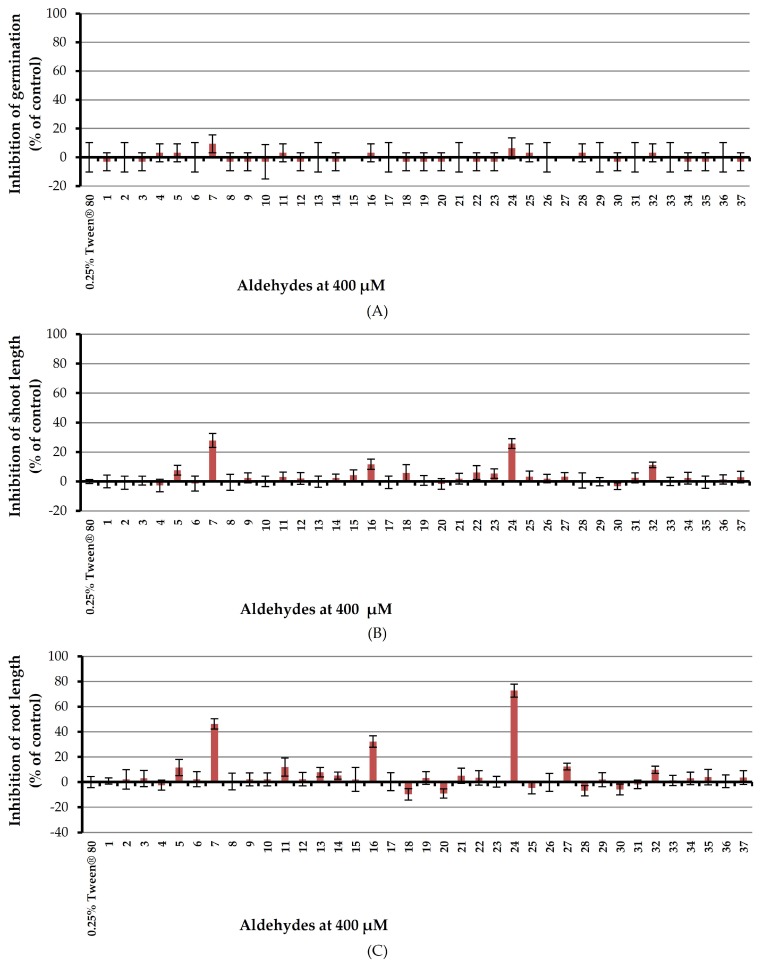
Inhibitory effects of aqueous solutions of thirty-seven aldehydes (at 400 µM) on seed germination (**A**), shoot length (**B**), and root length (**C**) of barnyardgrass. A 0.25% (*v*/*v*) aqueous solution of Tween^®^ 80 was used as a control.

Inhibitory effect of aldehydes at 400 μM on monocotyledon plant, barnyardgrass, was investigated by utilizing an aqueous solution of Tween^®^ 80 as a surfactant (Figure 6).

Results showed that all tested aldehydes had no effect on seed germination of barnyardgrass. In terms of shoot growth, (*E*)-cinnamaldehyde (**7**) and 4-(dimethylamino)benzaldehyde (**24**) moderately inhibited shoot length by 27.83 and 25.80% respectively but other aldehydes showed very low or no harmful effects. For root growth, (*E*)-cinnamaldehyde (**7**), 2,4,6-trimethoxybenzaldehyde (**16**), and 4-(dimethylamino) benzaldehyde (**24**) inhibited root growth of the tested plant by 46.20, 32.21 and 72.77% respectively but other aldehydes, again, had very low or no effects.

**Figure 7:**
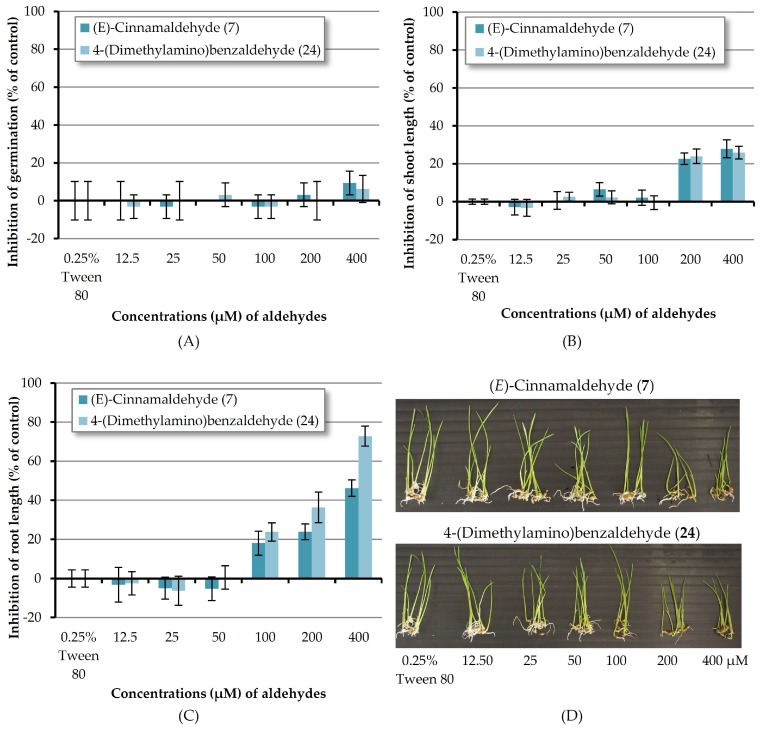
Inhibitory effects of aqueous solutions of two aldehydes (at 12.5–400 µM) on seed germination (**A**), shoot length (**B**), and root length (**C**) of barnyardgrass. A 0.25% (*v*/*v*) aqueous solution of Tween^®^ 80 was used as a control. (**D**) Shoot and root growth of barnyardgrass.

As mentioned above, (*E*)-cinnamaldehyde (**7**) and 4-(dimethylamino)benzaldehyde (**24**) are the most reactive chemicals toward barnyardgrass. In order to know the minimum concentration that the two compounds could inhibit the germination and seedling growth of a monocot plant; these two substances were then tested at concentrations of 12.5–400 μM (Figure 7). It revealed that at 200 μM aldehydes **7** and **24** inhibited shoot growth by 26.62 and 23.93% respectively but at other lower concentrations, both compounds showed no effect. In the case of root length, both chemicals could inhibit root growth at the concentrations down to 100 μM. Compound **7** and compound **24** at the concentration of 200 μM inhibited root length by 23.81 and 36.31% respectively and at the concentration of 100 μM inhibited root elongation by 18.00 and 23.72% respectively. However, at lower concentrations both compounds showed no inhibition.

The inhibitory effect of those thirty-seven aldehydes on dicot and monocot seeds has been unclosed in the present study. A comparison between the two species of plants showed that this group of chemicals tends to exhibit a stronger effect on Chinese amaranth than barnyardgrass. This is in agreement with our previous work [18] that we investigated the allelopathic effect of Makhwaen fruits on germination and growth of Chinese amaranth and barnyardgrass and eventually led us to isolate an active phenolic, xanthoxyline. After evaluation of the allelopathic activity of this compound on the tested plants we found that the germination of the dicot plant was totally inhibited at a concentration of 2,500 μM. However, at the same applied concentration, this compound showed a lower inhibitory effect on barnyardgrass. Furthermore, in the present study, most aldehydes affected root growth more than shoot growth and all of the tested compounds had no effect on seed germination of barnyardgrass. Similarly, in 2016, Gauda and coworkers [30] conducted a study on the herbicidal activity of a variety of monoterpenes against barnyardgrass. They found that, generally, these monoterpenes were more effective against seedling growth than seed germination of the plant. Besides, the inhibition of root development by all compounds was greater than that of shoot growth. Our results here indicated that 4-(dimethylamino)benzaldehyde (**24**) exhibited the greatest detrimental effect on the root length of barnyardgrass but this substance had a low inhibitory effect on the germination and seedling growth of Chinese amaranth. Also, the herbicidal potentials of the tested aldehydes relied on the applied concentrations. These suggested that the inhibitory effect of these chemicals is species-specific and concentration dependent.

## 3. Experimental

### 3.1. Chemicals

Tween^®^ 80, acetaldehyde (**1**), propionaldehyde (**2**), butyraldehyde (**3**), (*E*)-crotonaldehyde (**5**), (*E*)-cinnamaldehyde (**7**), *o*-tolualdehyde (**8**), cuminaldehyde (**9**), 2-(trifluoromethyl)benzaldehyde (**10**), 2-fluorobenzaldehyde (**11**), 2,4-dichlorobenzaldehyde (**12**), 3-bromobenzaldehyde (**13**), *p*-anisaldehyde (**14**), *m*-anisaldehyde (**15**), 2,4,6-trimethoxybenzaldehyde (**16**), vanillin (**17**), 3-nitrobenzaldehyde (**20**), 2-formylbenzoic acid (**21**), 4-formylbenzoic acid (**22**), 2-(2-formylphenoxy) acetic acid (**23**), 4-(dimethylamino)benzaldehyde (**24**), 4-((2-hydroxyethyl)(methyl)amino) benzaldehyde (**25**), 4-(*bis*(2-hydroxyethyl)amino)benzaldehyde (**26**), furfural (**28**), 1*H*-pyrrole- 2-carbaldehyde (**29**), thiophene-2-carbaldehyde (**30**), 1-methyl-1*H*-pyrrole-2-carbaldehyde (**31**), picolinaldehyde (**32**), 2-bromonicotinaldehyde (**33**), oxazole-4-carbaldehyde (**34**), thiazole-2-carbaldehyde (**35**), 1*H*-indole-3-carbaldehyde (**36**) and 1*H*-pyrrolo[2,3-*b*]pyridine-2-carbaldehyde (**37**) were purchased from Sigma-Aldrich (Singapore). Hexanal (**4**), benzaldehyde (**6**) and 4-hydroxybenzaldehyde (**18**) were purchased from Fluka (Buchs, Switzerland). Salicylaldehyde (**19**) and 4-morpholinobenzaldehyde (**27**) were purchased from Tokyo Chemical Industry (TCI, Tokyo, Japan). All compounds were reagent grade and used without further purification.

### 3.2. Preparation of Aqueous Solutions of Tween^®^ 80 at Concentrations of 0.06–1.00% (v/v)

As previously described [45], to a 100 mL-beaker, 1 mL of Tween^®^ 80 surfactant and 40 mL of distilled water were added. The mixture was well mixed by continuous stirring at room temperature for about 10 min. Then, the clear solution was transferred to a 100 mL–volumetric flask. Adjust the volume of the flask by adding distilled water, and followed by inverting the flask many times to obtain a 1% (*v*/*v*) Tween^®^ 80 stock solution. Other required concentrations were prepared by a dilution method to afford the aqueous solutions of Tween^®^ 80 at 0.50, 0.25, 0.13, and 0.06% (*v*/*v*) respectively.

### 3.3. Preparation of Aqueous Solutions of Aldehydes at 400 μM

Into a 100 mL-beaker, forty micromoles of a pure aldehyde and 0.25 mL of Tween^®^ 80 were added. The mixture was blended until it became clear (or no solid sample remains). To the mixture, 40 mL of distilled water was added, and the mixture was continuously stirred for 10 min. This thoroughly mixed solution was transferred to a 100 mL-volumetric flask and the volume of the flask was adjusted with distilled water to obtain a 400 μM of a pure aldehyde which contained 0.25% (*v*/*v*) of Tween^®^ 80 surfactant.

### 3.4. Preparation of Aqueous Solutions of Aldehydes at 400, 200, 100, 50, 25 and 12.5 μM

The 400 μM stock solutions of aldehydes **5**, **7**, **24** and **32** were prepared as described in Section 3.3. Aqueous solutions of compounds **5**, **7**, **24** and **32** at concentrations of 200, 100, 50, 25 and 12.5 μM were prepared by diluting the stock solutions with 0.25% (*v*/*v*) aqueous solution of Tween^®^ 80.

### 3.5. Tested Plants

Seeds of Chinese amaranth and barnyardgrass were used in the assessment of the herbicidal activity. Chinese amaranth seeds were purchased from Thai Seed & Agriculture Co. Ltd., Bangkok, Thailand, and barnyardgrass seeds were collected from rice fields in Phitsanulok Province, Thailand, in August 2016. The seed germination tests of both species were found to be >80%.

### 3.6. Seed Germination and Seedling Growth Bioassay

As previously described [45], to a small glass vial (4.5 cm × 2 cm) lined with germination paper, 0.5 mL of an aqueous solution of aldehydes were added. Ten seeds of a tested plant were then placed on the germination paper. The vials were sealed with Parafilm^®^ (in order that the solution does not dry out) and maintained at 28–30 °C in a growth chamber (cool white 840 Climacell 707, Munich, Germany). The chamber was set with a 12/12 h dark/light photoperiod, a light intensity of 100 µmol m^−2^·s^−1^, and around 80% of relative humidity. An aqueous solution of Tween^®^ 80 at a concentration of 0.25% (*v*/*v*) was used as a control experiment. The treatments and control group were replicated four times. After 7 days, numbers of seed germination were counted, shoot length and root length were measured, and percentages of inhibition were calculated as follows:

(1) $$Inhabition(\%\;of\;control)=100-\frac{\left(aldehyde\right)}{\left(control\right)}\times 100$$

### 3.7. Statistical Analysis

For the effect of Tween^®^ 80 on seed germination and seedling growth of barnyardgrass, a completely randomized design (CRD) was used. Data were subjected to the analysis of variance and comparisons were made between treatments at probability level *p* ≤ 0.05 using Tukey’s studentized range test.

## 4. Conclusions

In the present SAR study, the allelopathic effects of a variety of aldehydes were investigated by using Chinese amaranth as a representative of dicot plant and barnyardgrass as a representative of monocot plant. Factors determining the reactivity of those aldehyde allelochemicals were found to be unsaturation of structures, type, number and position of substituents and concentrations of aldehydes. Most aliphatic aldehydes had no allelopathic effect but α,β-unsaturated compounds showed supreme activity, especially the most reactive aldehyde, (*E*)-cinnamaldehyde (**7**) which could inhibit both dicot and monocot species. Aromatic aldehydes with methoxy-, hydroxyl- and alkylamino- substituents in the right position could also inhibit seed germination and seedling growth of the tested plants. Regarding heteroaromatic aldehydes, picolinaldehyde (**32**) impressively effected the germination and growth of the dicot plant in comparison with other related chemicals. Interestingly, 4-(dimethylamino)benzaldehyde (**24**) showed chemical clues suggesting a species-specific compound. Obviously, this substance highly inhibited root growth of barnyardgrass. Further research is still needed to find a mode of actions of these reactive aldehydes and also to develop potential natural product based herbicides in agrochemical industry.
